# Stereotactic Body Radiotherapy (SBRT) for Oligometastatic Spine Metastases: An Overview

**DOI:** 10.3389/fonc.2019.00337

**Published:** 2019-05-01

**Authors:** Kang Liang Zeng, Chia-Lin Tseng, Hany Soliman, Yonatan Weiss, Arjun Sahgal, Sten Myrehaug

**Affiliations:** Department of Radiation Oncology, Odette Cancer Centre, Sunnybrook Health Sciences Centre, Toronto, ON, Canada

**Keywords:** stereotactic body radiotherapy (SBRT), oligometastases, spine metastases, response assessment, outcomes, toxicities

## Abstract

The oligometastatic state is hypothesized to represent an intermediary state of cancer between widely metastatic disease and curable, localized disease. Advancements in radiotherapy have allowed for delivery of high precision, dose escalated treatment known as stereotactic body radiotherapy (SBRT) to targets throughout the body with excellent rates of local control. Recently, the first phase II randomized trial comparing conventional radiotherapy to comprehensive SBRT of oligometastatic disease demonstrated an overall survival and progression free survival advantage. The spine is a common site of metastasis, and a complex site for SBRT given the adjacent spinal cord and the tumor embedded within the bone tissue putting the patient at risk of fracture. Although there are expert spine SBRT guidelines for practice, there are as yet no reported randomized trials that proves superiority as compared to conventional radiation. The use of SBRT in patients with oligometastatic disease and spinal metastases is the focus of this review.

## Oligometastases and SBRT

Hellman and Weichselbaum first proposed the clinical *oligometastatic* state in 1995 to reflect a subset of patients with limited metastatic disease (1). From the spectrum theory, this is suggested to represent an intermediary cancer state where the biological profile of a cancer may not progress to widespread metastases (2). Within this group, an opportunity arises where targeted treatment toward limited metastases may confer disease and even possibly survival advantages. Advancements in imaging techniques (i.e., MRI, PET), and development of cancer specific imaging strategies (i.e., PSMA-PET), have allowed for greater ability to identify those with oligometastatic cancer.

Select patients with oligometastatic disease to the lung and liver are considered for surgical metastectomy and within this highly selected group, observed outcomes in a non-randomized setting were promising. The International Registry of Lung Metastases included 5,206 patients over five decades, and demonstrated 5-year overall survival (OS) of 36% after resection of limited lung metastases from mostly epithelial cancers or sarcomas (3). In colorectal patients, hepatic resection is considered for limited liver metastases with survival nearing 50% at 5 years (4).

Advancements in radiotherapy over the past decade, specifically in image-guided linear accelerator technology, treatment planning, and better understanding of normal tissue constraints with hypofractionated radiation, has led to increased interest in safe delivery of ablative doses of radiation with stereotactic body radiotherapy (SBRT). Advantages of SBRT in comparison to metastectomy includes the lack of surgical recovery time, side effect profile, and ability to safely target multiple metastatic lesions. SBRT may be secondarily advantageous in inducing an abscopal effect especially in malignancies strongly associated with an immune response (5).

**Figure 1:**
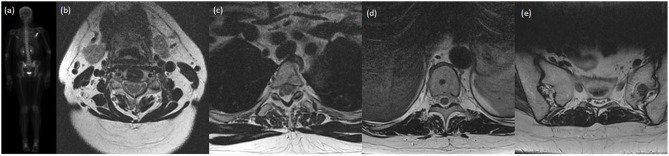
A case presentation of a lady with invasive ductal carcinoma of the breast who was treated definitively with lumpectomy, adjuvant chemotherapy, and adjuvant radiotherapy. Shortly after completion of therapy, on re-staging investigations, she was found to have oligometastatic disease in the bones, specifically at C4, T3, T10, the left sacral ala, and right scapula. She received SBRT to each site and was started on hormonal therapy. At most recent follow-up 20 months later, she has not had progression of known disease nor interval development on new metastatic disease. **(a)** Posterior-anterior projection of pre-treatment bone scan demonstrating increased uptake within the right scapula, T3 and the right sacral ala. Subsequent images of axial slice of T2-weighted MRI demonstrating near complete marrow replacement of C4 **(b)**, focal marrow abnormality in posterior T3 body **(c)**, rounded focus centrally of the T10 vertebral body **(d)**, and 13 mm lesion of left sacral ala **(e)**.

High quality evidence supporting the role of SBRT to oligometastases with traditional endpoints such as overall survival (OS) and progression free survival (PFS) are lacking, but a significant volume of researchers are attempting to answer this question. The SABR-COMET study was presented at the 2018 American Society for Radiation Oncology annual scientific meeting and represents the first Phase 2 randomized study to report improved outcomes in targeting oligometastatic disease with SBRT (6). This study included 99 patients randomized 1:2 to palliative standard of care (SOC) treatments vs. standard of care plus SBRT to all metastatic lesions (to a maximum of 5 lesions). Median overall survival was 28 months in the SOC arm compared to 41 months in the SBRT arm (*p* = 0.09) and PFS was significantly improved (6 months in the SOC arm vs. 12 months in the SBRT arm, *p* = 0.001). The results of confirmatory Phase 3 randomized studies such as CORE, SARON, and NRG-BR002 are eagerly awaited. A case demonstration of a patient treated under this approach is described in Figure 1. In the non-small cell lung cancer population specifically, two trials have assessed consolidative local therapy in oligometastatic disease (7, 8) with both noting significant improvements in progression free survival compared to maintenance therapy alone.

## Spine Metastases and SBRT

The spine is a common location for metastases and confers significant morbidity and mortality. The classical treatment approach for patients with symptomatic spine metastases is conventional palliative radiotherapy delivered with two parallel opposed beams with common fractionation regimens such as 8 Gy in 1 fraction, 20 Gy in 5 fractions, or 30 Gy in 10 fractions. Though effective in improving symptomatology, there is poor local control (LC) (9). With the availability of more lines of systemic therapy improving patient survival, there is a desire in select patients to improve durable LC and prevent neurologic compromise. Delivery of high biological effective doses (BED) of radiotherapy with SBRT precisely to the spine yields prolonged local control along with pain relief (Table 1). For those with oligometastatic disease, SBRT of known disease can prolong progression-free survival and potentially delay entry to next line of systemic therapy (29). In the post-operative setting, neurologic status is maintained through improvements in local control after SBRT. Further, following prior spine radiotherapy, it is a method of safely retreating the same or adjacent segments while minimizing dose to critical neurological structures.

**Table 1:** Outcomes after spine SBRT for *de novo* metastases.

**References**	**Patients/spinal segments (n/n)**	**Histology**	**Dose fractionation [dose (Gy)/fractions]**	**Follow-up duration (median, months)**	**Local control (time, if available)**	**Pain response**
Tseng et al. (10)	145/279	Mixed	24/2	15	90.3% (1-year) 82.4% (2-years)	NR
Azad et al. (11)	25/25	Mixed	15–25.5/1–5	18	84%	2/3 had pain relief
Anand et al. (12)	52/76	Mixed	24–27/1–3	8.5	94% (1-year) 83% (2-years)	90–94% complete pain relief
Bishop et al. (13)	285/332	Mixed	Median tumor dose 43 Gy (BED, a/b = 10)	19	88% (1-year) 82% (3-years)	NR
Sellin et al. (14)	37/40	RCC	24–30/1–5	49.0	57%	41% report pain improvement
Bate et al. (15)	24/24^*^	Mixed	16–30/1–5	9.8	96% (1-year)	NR
Sohn et al. (16)	13/13	RCC	38/4 (median)	NR	86% (1-year)	77% overall (23% complete pain response)
Guckenberger et al. (17)	301/387	Mixed	10–60/1–20	11.8	90% (1-year) 84% (2-years)	44% with severe pre-treatment pain, pain free. 56% with mild/moderate pre-treatment pain, pain free.
Thibault et al. (18)	51/51^*^	RCC	18–30/1–5	12.3	83% (1-year) 66% (2-years)	NR
Garg et al. (19)	47/47	Mixed	16–24/1	17.8	88% (18 months)	18 patients pain-free post-treatment compared to 13 patients pre-treatment
Chang et al. (20)	93/131	Mixed	NR	23.7	89% (1-year)	NR
Gill et al. (21)	14/14^*^	Mixed	30–35/5	34	80% (1-year) 73% (2-years)	NR
Wang et al. (22)	149/166	Mixed	27–30/3	15.9	81% (1-year) 72% (2-years)	54% pain free at 6-months, compared to 26% at baseline
Staehler et al. (23)	55/105	RCC	19–20/1	33.4	94% (1-year) 90% (2-years)	Median pre-treatment score 5, median post-treatment score 0 1 week after
Sahgal et al. (24)	14/18	Mixed	24/3 (median)	9	72%	NR
Yamada et al. (25)	93/103	Mixed	18–24/1	15	93% (2-years)	NR
Chang et al. (26)	17/22	Mixed	27–30/3–5	NR	68%	Narcotic usage fell from 60% at baseline to 36% at 6 months
Gerszten et al. (27)	8/8^*^	Breast	15–22.5/1	16	100%	Long-term axial and radicular pain improvement occurred in 96% who were treated primarily for pain
Ryu et al. (28)	49/61	Mixed	10–16/1	NR	96% (9-months)	Overall response 85%

NR, not reported;

* *Assuming one segment per patient*.

Specific to spine oligometastases, Barzilai et al. reported results from the AO Spine multicenter prospective cohort Epidemiology, Process, and Outcomes of Spine Oncology (EPOSO) study (30). Patients with oligometastatic disease (defined as <5 metastases) showed evidence of better survival compared to those with polymetastatic disease (>5 metastases). Of note, improved local control at 6 and 12 months were identified in the solitary/single spine metastasis subgroup, reflective of increased utilization of aggressive surgical and/or radiosurgery approaches.

Spine SBRT pertains unique considerations due to the balance of risk of neurologic compromise related to tumor progression and toxicities such as vertebral body fracture and myelopathy. Advancements in radiation planning and delivery, image guidance, robotic patient positioning, and understanding of dose tolerances to critical structures have made spine SBRT possible. With greater clinical experience, guidelines have been developed to direct safe practice (31–33) though supporting high-quality Phase 3 randomized data are pending. Delivery of spine SBRT requires careful patient selection, familiarity with the technique and an understanding of potential toxicities.

## Patient Selection

Compared to conventional external beam radiotherapy, spine SBRT is significantly more resource intensive from both a patient and systems perspective. Multidisciplinary discussion with specialized spine surgeons, radiologists, radiation, and medical oncologists is essential for careful selection of patients to avoid treatment of those that may not benefit. Practical considerations such as funding for novel techniques must also be considered, where “payers,” either that of public systems or private health insurance, may be reluctant to reimburse costly treatment modalities with limited prospective, high quality evidence justifying their use.

A number of schemes have been proposed to assist in identification of patients that benefit most from spine SBRT (34–36). Laufer et al. developed a four-point framework in the treatment of spine metastases (35). The Neurologic, Oncologic, Mechanics, and Systemic (NOMS) assessments assist in determining the optimal therapy for patients. The International Spine Oncology Consortium Report similarly proposes a multidisciplinary algorithm for the management of spine metastases given the recent advances in spine SBRT, and utilizes similar principles to guide management (34).

### Prognosis

Patients with spine metastases, despite being generally thought to be incurable, represent a heterogenous population (37) where some may live many years (i.e., a patient with oligometastatic hormone responsive prostate cancer) whilst others a significantly shorter time interval (i.e., one who has failed second line systemic therapy for widely metastatic pancreatic cancer). In the former case, one may consider more aggressive techniques such as SBRT, favoring long-term local control as this patient would derive most benefit, whereas the latter patient may benefit most from conventional palliative radiotherapy (38), or possibly best supportive care alone. One should identify patients with favorable prognoses who may derive benefit from spine SBRT. Age, performance status, comorbidities, and functional capacity can assist in determination of such. The prognosis of patients as predicted by physicians is often generous, however, specific to spine metastases, Jensen et al. propose a Prognostic Index for Spine Metastases (PRISM) which can assist in determining the most appropriate method of treating spine metastases (39). Briefly, scoring accounts for gender, performance status, previous therapy at the intended treatment site, number of organ systems involved, time elapsed between diagnosis and metastasis, and number of spine metastasis. The scoring system categorizes patients into groups 1 (best prognosis) through 4 (worst prognosis), with median overall survivals not reached in subgroup 1, and 24.1, 13.1, and 6.5 months in groups 2, 3, and 4, respectively.

### Histology

Histologies traditionally felt to be radioresistant (renal cell carcinoma, melanoma, sarcoma) demonstrate poor tumor control rates with conventional radiotherapy techniques (40, 41). Spine SBRT may overcome this radioresistance. In renal cell carcinoma specifically, local control at 1-year has been reported to be >80% (18, 42). As such, there is preference toward SBRT for patients with radioresistant histologies where local control is desired. In contrast, highly sensitive histologies, such as hematologic malignancies or small cell lung cancer may warrant upfront systemic therapy or derive similar benefit with conventional radiotherapy.

### Systemic Disease and Systemic Treatment Options

Assessment of systemic burden of disease and the availability and response to systemic therapies can influence patients' goals of care. In patients with widely metastatic disease, there may be an urgency to proceed with systemic therapy over focal treatment of minimally symptomatic spinal disease. Further, the availability of further lines of systemic treatment options is intimately related to prognosis, and clinicians may favor conventional techniques in those with high visceral burden of disease with limited further options or prognosis.

### Stability and Epidural Spinal Cord Compression

Mechanical spinal instability and presence of high-grade epidural spinal cord compression (ESCC) are independent indications for potential surgical intervention; radiotherapy, either with SBRT or conventional techniques may not be the most appropriate upfront in patients with reasonable prognoses.

Mechanical instability is usually not corrected with radiotherapy alone. As a method of grading instability, the Spinal Instability Neoplastic Score (SINS; Table 2) is a validated assessment tool of spine disease which may warrant surgical intervention (43–45). This score considers location, presence of mechanical pain, type of bony lesion, spinal alignment, vertebral body collapse, and posterolateral involvement and generates a score ranging from 0 to 18, with stable segment scores between 0 and 6, potentially unstable segments scoring between 7 and 12, and unstable lesions between 13 and 18. Potentially unstable and unstable lesions may warrant surgical evaluation.

**Table 2:** Spinal instability neoplastic score (SINS).

**Category**	**Description**	**Score**
Location	Junctional (occiput-T2, C7-T2, T11-L1, L5-S1)	3
	Mobile (C3-C6, L2-4)	2
	Semirigid (T3-T10)	1
	Rigid (S2-S5)	0
Pain	Yes	3
	Occasional non-mechanical pain	1
	No	0
Bone lesion	Lytic	2
	Mixed lytic/blastic	1
	Blastic	0
Alignment	Subluxation/translation	4
	*De novo* deformity	2
	Normal	0
Vertebral body	>50% collapse	3
	<50% collapse	2
	No collapse but >50% involvement by tumor	1
	None of the above	0
Posterolateral involvement	Bilateral	3
	Unilateral	1
	None	0

*The SINS score adapted from Fisher et al. (39).

In the case of epidural disease, the degree of ESCC and its potential consequences such as myelopathy or radiculopathy must be evaluated. Grading the severity of ESCC is commonly done via the Bilsky score, which facilitates communication between health-care providers (46). SBRT may be a more appropriate treatment option for those patients with appropriately graded low volume epidural disease. However, in the setting of acute clinical changes and/or high grade ESCC (Bilsky 2 or 3, and possibly 1c) patients warrant surgical evaluation. Consideration can be made to separation surgery, in which surgery to establish the epidural space is performed, followed by SBRT (47).

### Post-operative SBRT

High grade ESCC and/or mechanical instability often warrants surgical intervention in the appropriate patient population. In this setting, significant rates of local recurrence (up to 69.3% at 1-year) (48) justifies adjunctive therapies. Post-operative radiotherapy has traditionally been delivered with conventional techniques (49), although recently SBRT in this setting has been explored (50). Overall, post-operative SBRT was well tolerated [no grade 3 or 4 toxicities, 3.8% rate of grade 1/2 gastrointestinal and genitourinary toxicities, 9% rate of pain flare and vertebral compression fracture (VCF)] with excellent one-year local control between 84 and 88% reported (47, 51).

## Spine SBRT Technique

**Figure 2:**
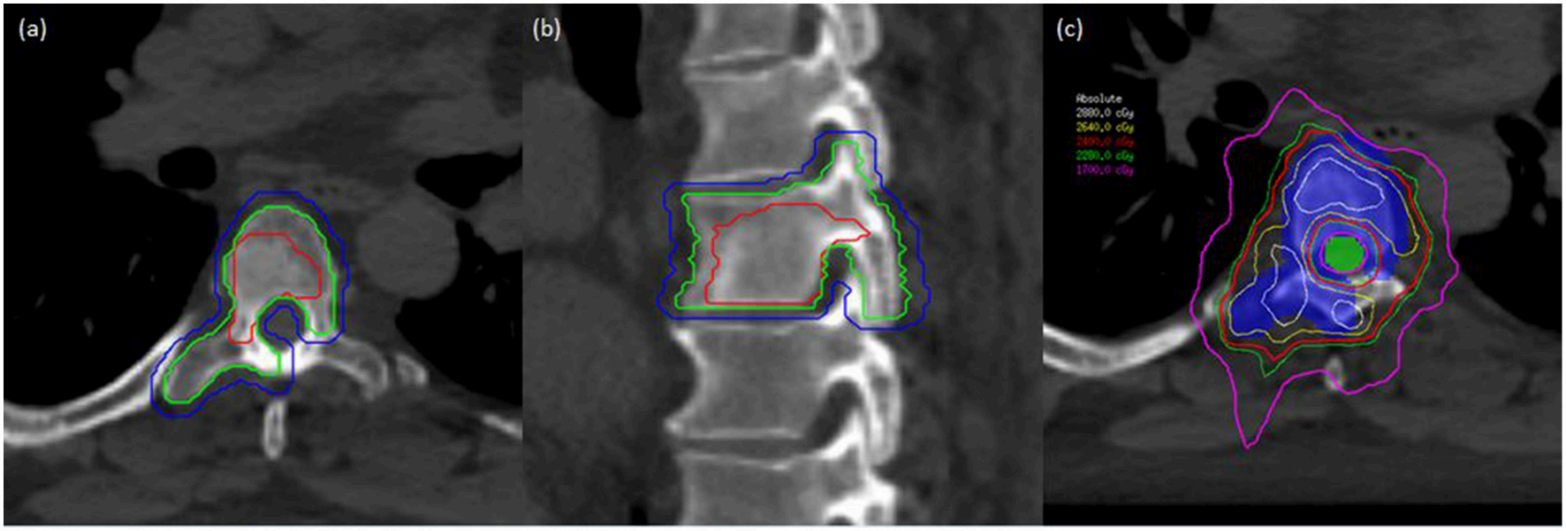
A man with oligometastatic castrate-resistant prostate cancer with painful spine metastases. This man was treated to 24 Gy in 2 fractions. **(a)** Axial planning CT scan demonstrating T6 vertebral level with gross tumor volume (GTV), clinical target volume (CTV), and planning target volume (PTV) delineated with red, green, and blue lines, respectively. **(b)** Sagittal planning CT demonstrating T6 vertebral level with GTV, CTV, and PTV in red, green, and blue, respectively. **(c)** Dose distribution at the level of T6 with PTV (colorwash blue) and spinal cord planning organ at risk volume (PRV) in colorwash green. Demonstration of sharp-dose fall-off to respect critical structures while allowing coverage of the target volumes.

Safe delivery of high doses of radiation to the spine is imperative to avoid potentially catastrophic neurologic sequelae. Recent advances in treatment planning, immobilization, treatment delivery and a better understanding of toxicities associated with SBRT have allowed for advancements within this field (Figure 2). Near rigid patient immobilization, consensus treatment volume definitions, and image-guidance are key for delivery of spine SBRT (52).

Near rigid patient immobilization is required to allow for inter-fraction reproducibility and minimize planning target volumes, to sculpt dose to intended targets and avoid neurologic toxicities. Many methods of immobilization have been explored which must consider patient comfort during relatively long simulation and treatment times. The physiologic motion of the spinal cord is < 0.5 mm in all directions (53), which is relatively insignificant compared to potential gross patient motion. Our practice is acquisition of a treatment scanning CT scan with patients secured using a BodyFIX device (Elekta AB, Stockholm, Sweden) which has demonstrated reproducibility within 1.2 mm and 0.9° with 95% confidence (52). Other immobilization devise include custom cradles (25) and stereotactic body frames (54).

Intra-fraction motion is a further consideration due to potentially long treatment times and patient comfort. Using either an evacuated cushion, vacuum body fixation or thermoplastic S-frame mask for lesions treated above T3, Li et al. performed pre-treatment verification cone beam (CBCT) as well as mid-fraction and post-treatment CBCT. The authors found margins required to encompass residual setup errors to be within 2 mm with vacuum body fixation and 3 mm with the other systems (55). Another study found a 3 mm planning margin to be sufficient to account for both intra-fraction and inter-fraction motion, with greatest intra-fraction motion in the x-plane of 0.7 mm (95% confidence interval 0.5–1.0 mm) (56).

After acquisition of planning CT scan, axial T1 and T2 weighted volumetric MRI sequences are fused to aid in target and critical neural structure delineation. In those cases where MRI is contraindicated or uninformative, CT myelogram may be an alternative.

The International Spine Radiosurgery Consortium has published consensus guidelines for target delineation in spine SBRT based on expert opinion with 10 representative cases (57). In general, gross tumor volume (GTV) should utilize all available imaging modalities and include epidural and paraspinal disease extension. The clinical target volume (CTV) should include areas of potential microscopic extension. In general, if GTV were present within the vertebral body, pedicle, transverse process, lamina, spinous process, the entire region should be included. In addition, as a rule of thumb, the adjacent potential bony region should be included. For example, GTV involving the vertebral body and right pedicle should correspondingly expand to a CTV encompassing the entire vertebral body, right pedicle, right transverse process and right lamina. With bone only disease, extraosseous expansion of CTV volumes should not be necessary, specifically into the epidural space or paraspinal soft tissue spaces. The planning target volume (PTV) was suggested to be a uniform expansion of ≤ 3 mm, depending on immobilization and image guidance technique.

In a separate study of post-operative epidural progression following SBRT, Chan et al. found that post-operative epidural disease extent underestimated treatment volumes and that consideration of pre-operative disease is crucial to prevent subsequent progression (58). An international group of experts led by Redmond et al. generated consensus contouring guidelines for post-operative spine SBRT (59). Recommendations were to include the entire pre-operative extent of both bony and epidural disease and immediately adjacent bony structures as part of the CTV. With circumferential epidural disease specifically, a “donut” shaped CTV was applied regardless of the post-operative epidural disease extent. Surgical instrumentation was suggested to be excluded from the CTV.

Optimal dose fractionation for spine SBRT is unknown. Common fractionation schemes include 16–24 Gy/1 fraction, 24 Gy/2 fractions, 24–30 Gy/3 fractions, 30 Gy/4 fractions, and 30–40 Gy/5 fractions. Considerations includes risk of vertebral compression fracture [up to 39% risk with single fractions (60)] and treatment volume, where very large tumors may warrant 4–5 fraction courses. Single fractions of 15 Gy are effective, however, may be related to increased toxicities such as VCF, pain flare and myelopathy, and fractionation may reduce this (61). Our standard practice is a course of 24–28 Gy in 2 fractions or 30 Gy in 4 fractions for larger tumors, to maintain an acceptable fracture risk of 10%.

There are differences in SBRT treatment planning compared to conventional techniques and Task Group 101 of The American Association of Physicists in Medicine outlines best practices (62). Perhaps the greatest change is allowing hotspots within treatment targets and the requirement for sharp drop-offs especially near organs at risk. As such, CTV and PTV margins are significantly smaller, whilst delivery with non-overlapping and possibly co-planer beams allow for sharp dropoff. Relating to spine SBRT specifically, there is an absolute requirement to not violate the thecal sac and spinal cord PRV dose limits for the sake of preventing catastrophic neurologic sequelae (63, 64). As such, it is acceptable for PTV coverage to be compromised.

Once a treatment plan has been generated, assessment of patient positioning on the treatment unit should be conducted. Image verification is completed with cone-beam CT after patient set-up. A Hexapod robotic couch (Medical Intelligence, Schwabmuenchen, Germany) facilitates set-up correction with six degrees of freedom. Subsequent CBCT can then be acquired for assessment of residual setup error, and intrafraction and post-treatment periods to ensure geometric stability. Other image verification techniques include CT-on-rails (65) and Cyberknife tracking (66).

## Outcomes

### Response Assessment

Assessment of response post-spine SBRT is challenging as criteria such as RECIST 1.1 are difficult to apply, and tumor specific phenomena exist whereby imaging must be interpreted with caution and with familiarity of expected changes after treatment. MRI signal changes creating a pseudoprogression phenomenon, as first seen following treatment of brain tumors, can occur after spine SBRT. Rather than true progression which demonstrates consistent growth over time, the radiographical appearance of pseudoprogression subsequently subsides on serial imaging. The incidence of pseudoprogression has been reported in the range of 14–37% and risk factors include lytic tumors, earlier volume enlargement, greater GTV to reference non-irradiated vertebral body T2 intensity ratio, and growth confined to 80% of the prescription isodose line (67–69).

In response to the need for common criteria assessing response post-spine SBRT, a group of international experts devised the SPIne response assessment in Neuro-Oncology (SPINO) guidelines as a method of standardized reporting (70). Recommendations of imaging response include spine MRI every 2–3 months for the first 12–18 months then every 3–6 months thereafter, interpreted by a radiologist and radiation oncologist jointly treating patients with this technique. Progression is defined as gross increases in tumor volume, new tumors in epidural space, and neurologic deterioration due to known epidural disease. Where progression is questionable, serial imaging and consideration of tissue biopsy should be made to rule out pseudoprogression. Assessment of pain response should be conducted with the Brief Pain Inventory at 3 months post-treatment adopting the consensus guidelines published by the International Bone Metastases Consensus Working Party (71).

### Local Control

Treatment of *de-novo* metastases with spine SBRT yields favorable local control, in the range of 80–95% in a heterogenous patient population, treated with a number of dose/fractionation regiments ranging from a single 15 Gy fraction to 30 Gy in 3 fractions (19, 22, 72). In a review of nearly 1,400 patients following SBRT, Hall et al. report overall local control of ~90% at 15 months (73). The largest single institutional experience utilizing 24 Gy in 2 fractions as standard for *de novo* metastases included 279 spinal segments from 145 consecutive patients (10). Local control at 1- and 2-years was 90.3 and 82.4% with excellent reported safety. There is a relative reduction in 2-year compared to 1-year LC ranging from 66 to 93% (Table 1). This may reflect the heterogenous nature of the mentioned studies, however merits further investigation. Though control rates at 2-years are still higher than with conventional palliative radiotherapy, in patients with limited metastatic disease and relatively excellent clinical status, durable LC is the treatment goal and endpoints beyond 1-year may be of further interest. In patients who do have local progression at this time point, retreatment with spine SBRT is safe and does offer excellent outcomes, though patients should be discussed in the multidisciplinary setting.

Retrospective studies have explored local control with a specific interest in traditionally radioresistant histologies that typically exhibit poor control with conventional external mean radiotherapy. One-year local control of 83% was reported after treatment of renal cell carcinoma (RCC) spine metastases treated with most common dose of 24 Gy in 2 fractions (18). Ghia et al. also report similar 1-year LC of 82% in RCC, and found that multi-fraction courses yielded inferior outcomes compared to single-fraction (sub-hazard ratio 6.57) which may suggest that BED escalation may be advantageous in radioresistant histologies (74). The high rates of local control are replicated in patients with sarcoma (75) and melanoma (76).

In the post-operative setting, inclusion of spine SBRT yields excellent local control, similar to *de-novo* metastases. Following vertebrectomy or laminectomy, 1-year LC in has been reported to be >80% in multiple studies (47, 77). In those where downgrading of epidural disease is surgically possible, local control is further improved (51). The considerations and treatment techniques are summarized in a critical review of post-operative spine SBRT by Redmond et al. (78).

Palliation of spine metastases with conventional techniques is limited by cumulative doses tolerated by the spinal cord. Despite high probability of pain response after conventional retreatment (79), local control remains poor which may become problematic for those with favorable prognoses. Especially in the modern setting of additional lines of systemic therapies that are potentially more efficacious, there is an increasing need to safely deliver retreatment to spine metastases. In a systematic review, local control after SBRT in this setting ranged from 66 to 90% at 1-year and improvement in pain scores post treatment ranged from 65 to 81% (80). Importantly, reirradiation was safe; vertebral fracture rate was 12% and treatment related myelopathy was 1.2%. Hashmi et al. pooled outcomes after retreatment with SBRT in 7 institutions (81). The median initial conventional radiotherapy delivered was 30 Gy in 10 fractions and 60% were re-treated with a single fraction SBRT. Local control remained excellent at 83% and importantly, there were no cases of radiation myelopathy after treatment of 247 spinal segments.

### Pain Response and Quality of Life

Overall pain response after conventional palliative radiotherapy is ~62% regardless of fractionation schedule, with complete response rates of 24% (38). The duration of response can be for months, with retreatment considered after 4 weeks, which may be effective despite initial non-response (82). In spine SBRT, complete pain response ranging between 46 and 92% have been reported (42, 83).

It is hypothesized that delivery of higher BED of radiotherapy to the spine may yield improved pain response. It is unclear the optimal dose fractionation for pain response specifically, and whether this technique offers improvements in pain response compared to conventional radiotherapy. Recently, Sprave et al. conducted a randomized phase II trial with the endpoint of pain-control, enrolling 55 patients treated with either SBRT (24 Gy in a single fraction) vs. 3D conformal radiotherapy to a dose of 30 Gy in 10 fractions (84). The authors assessed response using the parameters as established by the International Bone Consensus Working Party (71). There was a trend toward improved complete response at 3 months (43 vs. 17%, *p* = 0.0568) and at 6 months, rates of complete response were significantly higher in the SBRT group (53 vs. 10%, *p* = 0.0034). Responses were also more durable after SBRT. The vertebral compression fracture risk was 8.7% at 3 months and 27.8% at 6 months. There were no grade ≥ 3 adverse events reported. This continues to be assessed in the randomized phase II/III setting with the ongoing NCIC CTG SC.24 trial comparing conventional palliative radiotherapy to a standardized spine SBRT dose of 24 Gy in 2 fractions and RTOG 0631 comparing a single fraction of 16 Gy vs. conventional 8 Gy in 1 fraction (85, 86).

In a multi-institutional, international analysis of 387 spine segments treated with a median dose of 28 Gy in 3 fractions, over 40% of patients with severe pretreatment pain were pain free (definitionally a complete response assuming no increase in analgesic intake) at last follow-up with a median follow-up duration of 11.5 months (87). Pain improvement after retreatment with SBRT has similarly reported to be high (66).

Quality of life (QOL) is an important endpoint which is frequently assessed in addition to physical symptom outcomes and radiographic disease status. Sprave et al. assessed QOL using validated instruments including the EORTC QLQ-BM22, QLQ-FL13, and QSC-R10 and found that QOL was not worse after SBRT for spine metastases compared to conventional palliative radiotherapy (88). This endpoint will also be assessed in the ongoing NCIC CTG.SC24 phase II/III clinical trial.

### Predictors of Failure

Progression after spine SBRT is most common within the epidural space and may reflect the relative underdosing of tumor when intimate with thecal sac, or inherent biological aggressiveness of spine metastases with epidural components (51, 89). Al-Omair et al. found that surgical downgrading epidural disease extent resulted in improved local control prior to spine SBRT (51). Methods of mitigating this influence on local control include considering escalating the allowable dose to the spinal cord, or interventional surgical techniques to target epidural disease extension.

## Toxicities

Spine SBRT is generally well-tolerated, and typically a threshold of <5% is accepted as risk of serious adverse events such as myelopathy. VCF rates have been relatively well-studied after spine SBRT, and a greater understanding of pretreatment assessment and radiotherapy technique has mitigated this risk.

### Pain Flare

Defined as a transient increase in pain shortly after commencing or completing radiotherapy, pain flare is common in approximately a third of patients after conventional palliative radiotherapy (90). The range of patients developing pain flare after spine SBRT is significant, from 14 to 68% (91–93). Dexamethasone has been prospectively evaluated in the prevention of pain flare and reduced its rate from 68 to 19% (94).

### Vertebral Compression Fracture

Delivery of a high BED of radiotherapy generates an intense acute inflammatory effect that is hypothesized to weaken the bony matrix and place patients at risk of VCF (60). The rate of VCF in the range of 11–39% with a crude risk of 13.9% in a review (60, 95, 96), compared to 3% for conventional radiotherapy (97). Regardless of the mechanism of VCF, both pre-treatment characteristics and treatment related parameters influence the rate of VCF that can result in further pain, and requirement for surgical stabilization. Median time to development of VCF was 2.5 months in a multi-institutional study including 57 fractures (98).

In retrospective analyses, the aforementioned SINS score includes several elements predictive of VCF including baseline fracture, lytic disease, spine malalignment, >50% vertebral involvement and the overall high SINS score was similarly predictive (60). Lee et al. assessed the capability of SINS in predicting fracture, and found that those in the high SINS group to have a 66.3% risk of fracture at 24 months compared to 21.3% for the low SINS group (99). Further, volume of lytic disease, a refinement of the SINS component, has independently been demonstrated to predict for SBRT-inducted VCF (100). These data support multidisciplinary assessment of patients with spinal metastases, especially in those with intermediate/high SINS scores who may benefit from surgical or minimally invasive procedures to stabilize the spine prior to radiotherapy.

High dose, single-fraction SBRT has been associated with a higher rate of VCF. Those receiving a single fraction of ≥24 Gy, compared to those receiving 20–23 Gy and those receiving ≤20 Gy had a 39% vs. 23% vs. 11% risk of fracture, respectively. In support of this, Rose et al. report a fracture rate of 39% after single doses ranging from 18 to 24 Gy (96). Our institution has observed an 8.5% 1-year VCF risk utilizing our standard 24 Gy in 2 fraction technique.

Sprave et al. assessed bone mineral density as a prespecified secondary endpoint in their study comparing conventional palliative radiotherapy to spine SBRT (101). Both conventional radiotherapy and SBRT increased bone mineral density at 3- and 6-months with one technique not being statistically significantly better. In osteolytic metastases specifically, SBRT increased bone density whereas conventional RT did not. These findings support the safety of spine SBRT, especially where vertebral body fracture is a consideration.

### Myelopathy

Radiation myelopathy is a late complication of SBRT and most feared due to potential catastrophic outcomes. A review of nearly 1,400 patients reveal that rates of myelopathy to be 0.4% (73). Point max doses to the spinal cord categorized by number of fractions was reported in a study of nine cases of myelopathy compared to 66 cases without by Sahgal et al. (102). With two fractions, a point max dose of 12.5, 14.6, 15.7, 16.4, and 17.0 Gy yielded an estimated risk of 1, 2, 3, 4, and 5% of myelopathy, respectively. In the reirradiation setting, after conventional external beam radiotherapy, a cumulative thecal sac point maximum dose of 70 Gy in equivalent 2 Gy per fractions (utilizing an alpha-beta ratio of 2) was suggested as long as sufficient time had elapsed since initial treatment (≥5 months) and the point maximum for retreatment should not exceed 25 Gy in equivalent 2 Gy fractions (101).

## Conclusions

The recent, first randomized clinical trial demonstrated overall and progression free survival benefits after SBRT to oligometastatic disease which supports prior retrospective case series (6). The spine is a common site of metastatic bone disease, and as high quality data continue to mature, along with completion of additional randomized clinical trials, it is expected that utility of SBRT to the spine will increase in the future.

Spine SBRT is unique due to the requirement of sharp dose falloff to prevent serious neurologic morbidity. With recent advances in radiotherapy planning, robotic patient positioning, image guidance and radiotherapy delivery, this has been made possible. Local control is excellent, and pain response is comparable to conventional radiotherapy. Patient selection is of utmost importance due to this resource intensive technique, and multidisciplinary consultation is warranted.

## Author Contributions

KZ, AS, and SM were responsible for the conception of this review. KZ and SM were primarily involved in the abstraction and analysis of data. All authors contributed to the writing of this review, editing, and final approval prior to submission.

### Conflict of Interest Statement

The authors declare that the research was conducted in the absence of any commercial or financial relationships that could be construed as a potential conflict of interest.
